# Three-Decade Evaluation of Cerebrospinal Fluid Pressure in Open-Angle Glaucoma at a Tertiary Care Center

**DOI:** 10.1155/2020/7487329

**Published:** 2020-11-11

**Authors:** Catherine G. Knier, David Fleischman, David O. Hodge, John P. Berdahl, Michael P. Fautsch

**Affiliations:** ^1^Mayo Clinic Alix School of Medicine, Mayo Clinic and Mayo Foundation, Rochester, MN, USA; ^2^Department of Ophthalmology, Mayo Clinic and Mayo Foundation, Rochester, MN, USA; ^3^Department of Ophthalmology, University of North Carolina, Chapel Hill, NC, USA; ^4^Department of Health Sciences Research, Mayo Clinic and Mayo Foundation, Jacksonville, FL, USA; ^5^Department of Ophthalmology, Vance Thompson Vision, Sioux Falls, SD, USA

## Abstract

Elevated intraocular pressure (IOP) is the most prevalent risk factor for primary open-angle glaucoma. However, IOP alone does not fully describe a mechanical basis for disease in patients with normal tension glaucoma or primary open-angle glaucoma. The translaminar pressure difference (TLPD) theory proposes that the pressure gradient generated by the difference of IOP and cerebrospinal fluid pressure (CSFp) acting at the level of the optic nerve can lead to cupping and glaucoma when IOP is higher than normal and/or CSFp is lower than normal. The study results to date have generally supported the TLPD theory; however, varying methods, populations, and sample sizes make it difficult to compare results. To further assess whether there is an association between low CSFp and open-angle glaucoma, 30 years of clinical data that assess 96,543 lumbar punctures were analyzed. Patients with open-angle glaucoma showed a significantly lower CSFp than randomly selected normal control patients (9.9 ± 3 mm·Hg (*n* = 86) versus 12.1 ± 3.6 mm·Hg (*n* = 114), *p* < 0.001) following adjustment for age and sex. This retrospective study provides strong evidence for an association between open-angle glaucoma and low CSFp.

## 1. Introduction

Elevated intraocular pressure (IOP) is the most prevalent risk factor for primary open-angle glaucoma (POAG), an optic neuropathy that tends to affect older patients [1]. The mechanical theory of optic nerve degeneration suggests that as IOP increases, a force is generated posteriorly inducing damage to retinal ganglion axons, which can be visualized as cupping on ophthalmic examination [1, 2]. However, not all patients with ocular hypertension (OHT) develop glaucomatous damage. Additionally, up to 90% of open-angle glaucoma (OAG) patients from certain Asian populations present with normal IOP readings (<22 mm·Hg; referred to as normotensive glaucoma (NTG)) even as glaucomatous damage progresses [1, 3]. The pathophysiology remains a mystery for these forms of glaucomatous disease. IOP elevation in the absence of glaucomatous damage (OHT) and, conversely, glaucomatous damage in the absence of IOP change (NTG) suggest the mechanical theory of IOP-induced glaucomatous damage is incomplete.

One complementing theory first posited by Noishevskiĭ in 1908 at the Conference of the Military Medical Academy in St. Petersburg, Russia, has gained support over the past decade. This theory posits that an imbalance in the pressure gradient across the optic nerve head, rather than IOP alone, generates a force that causes the optic disc to cup [4–6]. This pressure gradient, also called the translaminar pressure difference (TLPD), is hypothesized to cause damage to retinal ganglion axons by inhibiting axonal transport resulting in glaucoma. In simplified terms, the TLPD is the difference between IOP and the cerebrospinal fluid pressure (CSFp) within the optic nerve subarachnoid space. Thus, glaucomatous damage could be caused by an increase in IOP, a decrease in intracranial pressure, or both. This theory offers a common hypothesis for how NTG, POAG, and OHT may occur.

Over the last decade, a variety of prospective and retrospective studies have examined the TLPD theory in NTG and POAG. Studies using lumbar puncture or transcranial Doppler ultrasound to measure CSFp have found a reduced TLPD in NTG compared with POAG and control groups [7], with some noting a significant difference between POAG and controls [8–10]. At the same time, higher TLPD in NTG patients was found to correlate with a reduced neuroretinal rim area, providing further support for the TLPD theory [7]. Other studies measuring optic nerve subarachnoid space or flow as a surrogate for CSFp in living patients have also observed changes that correspond to an increased TLPD or decreased CSFp in NTG patients compared with POAG and controls [11, 12]. In one study, a cadaveric human eye was manipulated to change the CSFp at the optic nerve head while holding a constant IOP with results demonstrating CSFp independently modulates the strain measured at the optic nerve head, supporting a mechanical basis for the development of glaucoma in NTG patients due to TLPD dynamics [13]. In contrast, studies measuring the optic nerve sheath diameter as a surrogate for CSFp did not find a significant association with TLPD in OAG [12, 14]. Studies using measured or estimated CSFp based on patient BMI, diastolic blood pressure, and age have shown both positive and negative associations between CSFp, OAG, and OHT [8, 10, 15–17].

While results to support the TLPD theory have been generally consistent, the majority of previous studies utilized varying methods and small sample sizes, making it difficult to compare results. To address the association between CSFp and OAG, we evaluated 3 decades of clinical data, including a meta-analysis of one group from 1985 to 2006 and another from 2007 to 2019. This study achieved the appropriate sample size to address the question of whether OAG is associated with CSFp measured by lumbar puncture in a population of patients at a tertiary care center.

## 2. Methods

### 2.1. Ethics Approval

This study was approved by the Mayo Clinic Institutional Review Board (IRB #10-001493) and carried out in compliance with national and international ethical standards.

### 2.2. Lumbar Puncture

This study utilized inclusion and exclusion criteria over the period from 1985 to 2019 to replicate the analysis that was reported in previous studies [9]. Patients eligible for this observational case-control study were identified using Mayo Clinic's institutional data repository to identify individuals who received a lumbar puncture between 1/1/1985 and 3/31/2019. Lumbar punctures were performed in the manner previously described [10]. Briefly, the procedure was administered by a small team of registered nurses making up the lumbar puncture team with patients in the lateral decubitus position.

### 2.3. Selection of Patients

Patients with relevant ICD-9 and ICD-10 codes were used to generate case and control groups. Cases included patients with a diagnosis of POAG (365, 365.10, 365.11), NTG (365.0, 365.12), and OHT (365.04). Herein, OAG refers to the group including both POAG and NTG cases. Controls were chosen from patients with presbyopia, myopia, hyperopia, or senile cataract (366.10, 366.14, 366.15, 366.16, 366.17, 367.0, 367.1, 367.4). Along with ocular diagnosis, clinical data including the date of lumbar puncture, birth date, ethnicity, race, and biological sex were recorded. The medical record was reviewed to collect the indication for lumbar puncture, opening pressure, CSF protein, and glucose. At that time, ocular diagnoses were validated against the medical record. Additional data recorded included medications know to affect IOP (including beta blockers, prostaglandin analogues, alpha agonists, carbonic anhydrase inhibitors, or prednisolone), glaucoma or other ocular surgery history, systemic medications (including diuretics, systemic beta blockers, ACE inhibitors, other class hypertensive agents, and prednisone), presence of hypertension, diabetes, or stroke in the patient history, and blood pressure at or before time of the lumbar puncture. BMI was recorded for the 2007–2019 group.

### 2.4. Inclusion and Exclusion Criteria

The inclusion criteria consisted of patients greater than 30 years of age with an ophthalmic exam within 1 year of lumbar puncture. If the patient had multiple ophthalmic exams, the exam closest in time to the lumbar puncture procedure was used. Information from the closest exam required for inclusion was the patient's intraocular pressure (IOP) measurements taken by handheld and Goldmann applanation tonometry at Mayo Clinic and cup-to-disc ratios (C/D). In addition, the highest IOP for each eye and the highest combined IOP were recorded along with the respective exam dates. IOP measurements taken in the 2 weeks following ocular surgery were not used. In patients with a single ophthalmic exam on record, those IOP values were used as both the closest and the highest recorded values.

Stringent exclusion criteria were employed to rule out patients with abnormalities that may affect the lumbar puncture opening pressure. This included patients who did not have an eye exam within one year of the lumbar puncture date, any past or future medical or surgical history of a condition or treatment known to affect the intracranial pressure, any history of systemic carbonic anhydrase inhibitors, who had any eye condition with the potential to affect IOP or optic nerve integrity, if the complaint prompting the closest vision exam had to do with the indication for lumbar puncture, if the lumbar puncture was due to a vision loss or vision change symptom, an opening pressure outside the normal range (60–250 mm·H_2_O where 13.6 mm·H_2_O = 1 mm·Hg), or abnormal results of the CSF examination as interpreted by a board-certified neurologist.

### 2.5. Statistics

The sample size to achieve 80% power with *p* < 0.05 based on Berdahl et al. (2008) (mean and standard deviation for POAG CSFp 9.6 ± 3.1 mm·Hg; for age-matched controls 12.7 ± 3.9 mm·Hg) was calculated to be *n* = 48 in each group [10]. The control groups for OAG and OHT were randomly selected from the control cohort that met inclusion and exclusion criteria. Because OAG generally occurs in older adults, while OHT can occur in younger adults, the OAG control group was age-matched by randomly selecting from patients at least 55 years of age and the OHT control group from patients at least 30 years of age. Initial comparisons between glaucoma groups and controls were completed with a two-sample *t*-test. Categorical factors were compared between groups using Fisher's exact tests. Overall correlations between continuous variables were presented using Pearson correlation coefficients, and linear regression analysis was used to adjust for age and sex when evaluating those relationships. A *p*-value ≤ 0.05 was used to indicate significant differences. All analyses were completed using SAS, version 9.4 (Cary, NC).

## 3. Results

From 1985 to 2019, a total of 96,543 lumbar punctures were performed at Mayo Clinic. Among these, 382 unique subjects had a diagnosis of OAG, which include both POAG and NTG patients; 215 OHT; and 3,931 presbyopia, myopia, or senile cataract. From each group, the number of patients passing inclusion and exclusion criteria for this study was 86 with OAG (68 POAG, 18 NTG) and 44 OHT (Table 1). A total of 114 patients were included in the control group for OAG and 87 in the control group for OHT. Within this sample of patients, there was a significant difference in age between OAG cases (71.4 ± 12.7 years) and OAG controls (68.2 ± 8.4 years) (*p* = 0.04), but not OHT patients (56.9 ± 17.4 years) and OHT controls (57.2 ± 12.8 years) (*p* = 0.92). Sex was similar between cases and controls (OAG cases 41% female, OAG controls 44% (*p* = 1.0); OHT cases 53% female, OHT controls 50% (*p* = 0.69)).

**Figure 1:**
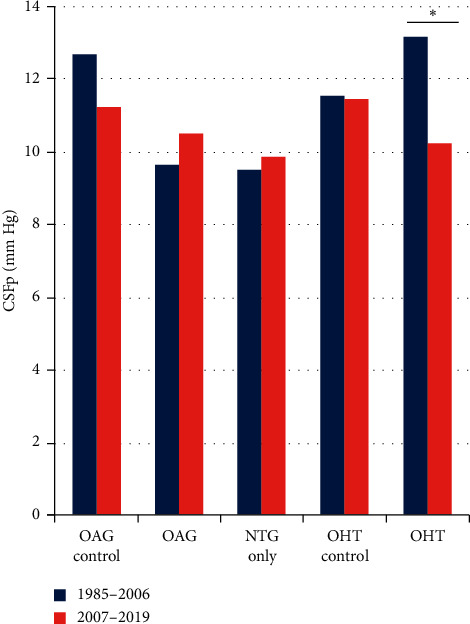
Validating the 2007–2019 data set against the 1985–2006 data set. There was no significant difference between the CSFp for OAG and OAG controls in the 2007–2019 data set (*p* = 0.24). The CSFp values were statistically similar between 1985–2006 and 2007–2019 data sets for the OAG and OAG control groups (1985–2006 OAG vs. 2007–2019 OAG, *p* = 0.29; 1985–2006 NTG vs. 2007–2019 NTG, *p* = 0.78). The CSFp value for OHT cases was significantly lower in the 2007–2019 data set than the 1985–2006 set. OAG, open-angle glaucoma; CSFp, cerebrospinal fluid pressure; NTG, normotensive glaucoma; OHT, ocular hypertension. ^*∗*^*p* = 0.009.

The most recent decade of data (2007–2019) were validated for consistency. The first 2 decades of this study have been previously validated [5, 9, 10]. The recorded demographic factors including ethnicity, race, and BMI were statistically similar between cases and controls in this period (Table 2). The mean age at time of lumbar puncture was higher in cases compared with controls for OAG and NTG only (Table 2). Accordingly, age was controlled in multivariate analysis. There were differences present in the indication for lumbar puncture between OAG cases and controls; however, CSF protein, glucose, systolic and diastolic blood pressure, systemic medications, and disease status were statistically similar (Table 2). Further investigation revealed a significant difference in CSFp between the subset of OAG patients referred for IRB-approved research purposes (8.8 ± 2.5 mm·Hg) versus all other indications (11.6 ± 3.0 mm·Hg) (*p* = 0.01). There was no significant difference in CSFp for any other single indication compared with the overall sample in the OAG, OHT, or control groups. The CSFp from the most recent decade compared with the two previous decades was not statistically different between the OAG groups (10.5 mm·Hg (2007–2019) and 9.6 mm·Hg (1985–2006), *p* = 0.29). There was a statistically significant difference between the CSFp of the OHT groups from the two time periods (10.2 mm·Hg (2007–2019) vs 13.2 mm·Hg (1985–2006), *p* = 0.009) (Figure 1).

**Figure 2:**
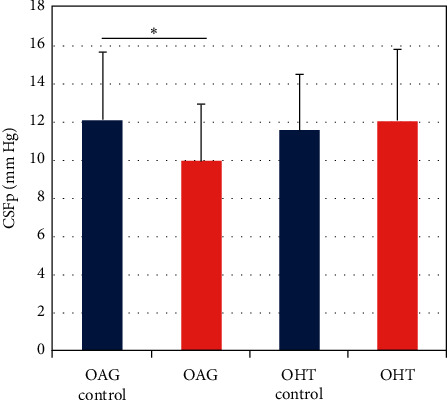
CSFp trends from 1985 to 2019. There is a significant difference in CSFp between OAG patients and controls. There is not a significant difference in the CSFp of OHT patients and controls. CSFp, cerebrospinal fluid pressure; OAG, open-angle glaucoma; OHT, ocular hypertension. ^*∗*^*p* < 0.001.

From 1985 to 2019, CSFp was significantly lower in OAG cases (9.9 ± 3.0 mm·Hg, *n* = 86) than OAG controls (12.1 ± 3.6 mm·Hg, *n* = 114) (*p* < 0.001) (Figure 2). This relationship persisted in multivariate models adjusting for age and sex (*p* < 0.001). In contrast, ocular hypertension patients (12.0 ± 3.8 mm·Hg, *n* = 44) did not differ from controls (11.5 ± 3.0 mm·Hg, *n* = 87) in a univariate comparison (*p* = 0.34) or in a multivariate analysis accounting for age and sex (*p* = 0.35).

**Figure 3:**
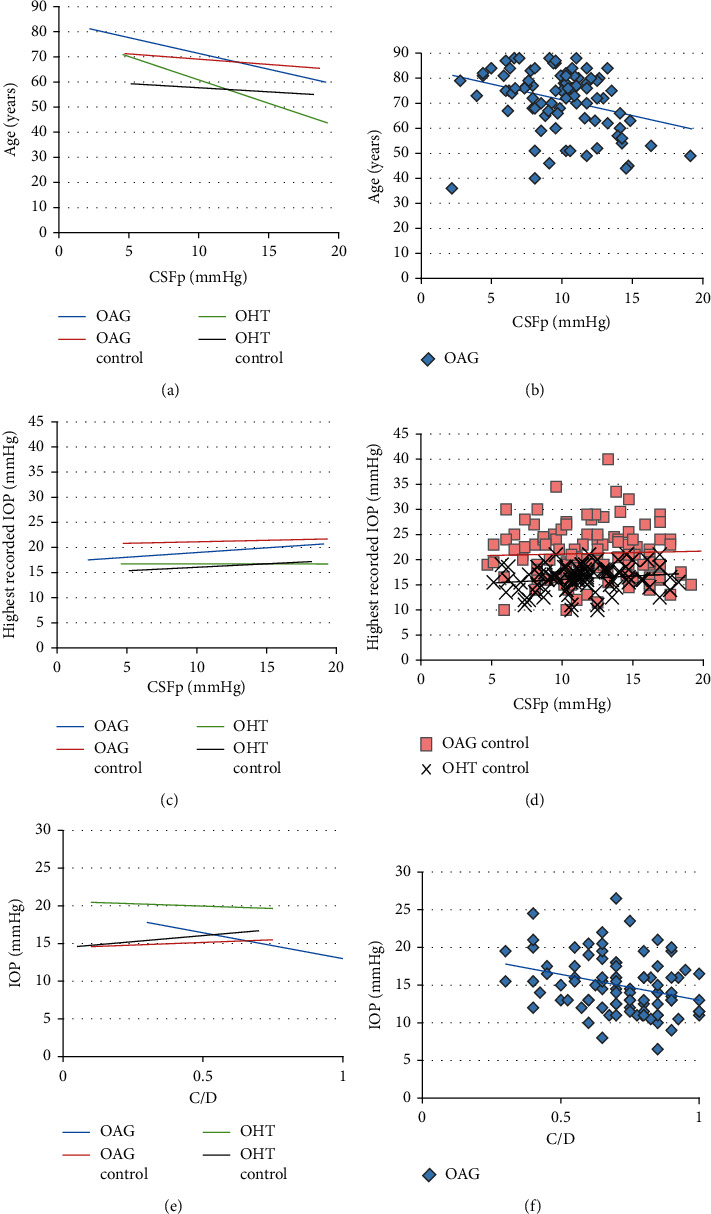
Linear regression analysis. (a, b) Analysis of age vs. CSFp showed a significant negative correlation in the OAG group. There was a negative relationship in all groups, but only the OAG group achieved significance. (c, d) Analysis of the average highest recorded IOP vs CSFp showed a significant positive correlation in both control groups. Significance was not achieved in either OAG or OHT cases. (e, f) Assessment of the IOP recorded closest to the date of lumbar puncture vs C/D showed a significant negative correlation in the OAG group. OHT trended towards a negative relationship, while controls trended towards a positive correlation, but none of these achieved significance.

Similar to studies previously reported in the literature, age was inversely related to CSFp in the OAG group (*r* = −0.31, *p* = 0.004) (Figures 3(a), and 3(b)) [18, 19]. The highest recorded IOP in controls, but not OAG or OHT cases, positively correlated with CSFp (OAG controls *r* = 0.31 (*p* = 0.001) vs OAG *r* = 0.01 (*p* = 0.97); OHT controls *r* = 0.27 (*p* = 0.02) vs OHT *r* = 0.01 (*p* = 0.97)) (Figures 3(c) and 3(d)). In addition, the closest IOP measurement demonstrated a negative relationship with respect to the C/D ratio in OAG cases, but no other groups (*r* = −0.24, *p* = 0.02) (Figures 3(e) and 3(f)). No statistically significant relationship was identified in the other groupings or variables.

## 4. Discussion

While several mechanistic theories have been hypothesized to account for the development of optic neuropathy and glaucoma, only the TLPD theory explains how glaucomatous conditions result in patients with POAG, NTG, and OHT. In POAG, patients with elevated IOP with or without decreased CSFp may develop degeneration of retinal ganglion cells due to an elevated TLPD. In NTG patients with normal IOP and retinal ganglion cell loss, the TLPD theory suggests that lower CSFP may exaggerate the balance between IOP and CSFp, resulting in optic nerve cupping. While the cause of lower CSFp is currently unknown, it is interesting that CSFp decreases with age starting at age 50, which is the same age that incident cases of NTG increase [5, 18]. These correlative studies suggest that as CSFp decreases with age, the TLPD (IOP-CSFp) increases and favors posterior cupping of the optic disc. Thus, glaucomatous damage occurs without altering the observed IOP. In OHT, where IOP is elevated but no retinal ganglion cell damage is observed, the TLPD theory would reason that an elevated CSFp keeps the TLPD in a normal range. In the current study, evidence from over three decades supports the relationship between OAG status and decreased CSFp, even in models adjusting for age and sex. In contrast, there is not an observable difference in CSFp between OHT patients and their respective controls. These results support the theory of TLPD as a major driver of OAG, rather than IOP alone. The results for OHT in this study are mixed. In contrast to prior studies, the lack of a relationship between increased OHT and increased CSFp calls into question the potentially counterbalancing effect of increased local CSFp and/or the role of CSFp in OHT itself. At the same time, the significant positive correlation between maximum IOP and CSFp in controls, but not OHT patients, suggests an equilibrium in healthy states that may be absent in OHT. With increasing acceptability of LP procedures in clinical study, further studies to determine patterns of CSFp dynamics in OHT are needed.

The observation of low CSFp in POAG and NTG patients can have major implications for the understanding, diagnosis, and management of OAG. IOP, CSFp, and blood pressure appear to exist in balance with one another, but the order of events leading to glaucomatous damage is uncertain [4, 20, 21]. It is important to note that the TLPD theory as the major mechanical driver of OAG does not preclude the importance of vascular pathophysiology. Vascular changes [17] may work in concert with the TLPD and other risk factors that affect glaucoma development and progression in the clinical setting. Ultimately, this work may help direct the development of novel interventions targeting the mechanical component of TLPD imbalance at the optic nerve head [22] and noninvasive, low-cost measures of CSFp that can be used in the clinical setting to improve early diagnosis of OAG, especially NTG [23, 24].

In contrast to the OAG group, the OHT group remains underpowered after three decades of study. The relationship of CSFp with OHT reported in the literature has been variable, some reports showing higher CSFp in this patient population [8, 9] and others showing no meaningful relationship. Thus, the possibility remains that the sample presented in this study does not represent the true relationship of OHT with CSFp in the population. It is also possible that CSFp is spuriously related to the disease mechanism. It would be valuable to conduct a properly powered study of OHT patients to determine whether or not there is a relationship with CSFp.

Linear regression performed to determine the presence or absence of relationships between clinical variables and eye-related variables in the overall time range (1985–2019) was consistent with previous findings [25]. Age had a significant negative relationship with respect to CSFp in the combined OAG group (as age increases, CSFp decreases). Interestingly, in both control groups, there was a positive linear relationship between the highest recorded IOP and CSFp. This relationship was not present in patients with OAG or OHT. This association may represent the normal dynamics between CSFp and IOP at equilibrium in healthy eyes and support the TLPD theory of a balance between CSFp and IOP in normal eyes. In OAG patients, IOP lowering therapies likely contribute to the alteration of IOP and CSFp dynamics observed as a significant negative relationship between IOP and the closest measured IOP. This could possibly be due to more aggressive IOP lowering therapy in patients with more severe clinical glaucoma and highlights the importance of identifying the maximum IOP in such studies. Further prospective studies that can measure LP and IOP on the same day will help to determine how TLPD is affected [8].

Analysis of the most recent decade of data alone (2007–2019) during validation showed a trend towards decreased CSFp in OAG patients compared with controls that were nonsignificant (*p* = 0.24). Considering the CSFp from this decade compared with those previously studied, it seems likely that this discrepancy is due to limited sample size. Taken in combination with achieving the necessary sample size in the 1985–2019 cohort and the strong statistical significance of the overall result increases confidence in the conclusion that CSFp is decreased in OAG patients compared with controls (*p* < 0.0001).

## 5. Limitations

From the most recent decade (2007–2019), the CSFp was significantly lower in the OAG group for those with IRB as an indication for lumbar puncture versus all other indications (*p* = 0.01). This was not the case for other groups or the other top 4 indications (headache, peripheral neuropathy, radiculopathy, and dementia). Even though the study design excludes individuals with diagnoses or medications known to alter CSFp, there may be confounding factors that are unknown in OAG patients that secondarily alter CSFp. For example, 13 (45%) OAG patients in this study who had undergone lumbar puncture for IRB research may be less psychologically and physiologically stressed than their peers receiving LP to understand pathology, leading to the lower CSFp observed in the IRB group. As lumbar puncture procedures appear to be increasingly accepted in otherwise normal patients for research purposes, an appropriately powered and well-controlled prospective study would be beneficial to improve the generalizability of this conclusion and reflect trends closest to the otherwise normal population of OAG patients. While significant measures were taken to ensure consistency between research methods across the three decades, there is always a chance that a difference between researchers could produce unintentional bias in the sample. Due to the consistent result of our overall data set, despite decade-to-decade differences, along with ample sample size across the three decades, we believe the observation that glaucoma is associated with decreased CSFp is reliable for this population of OAG patients presenting to a tertiary care center.

Another potential limitation is that the data are reporting lumbar CSFp measurements. While the lumbar CSFp may represent the intraventricular CSFp while the patient is in the lateral decubitus position [26], it likely does not represent the orbital CSFp, which is the most important measurement for determining the TLPD [27, 28]. CSF dynamics within the orbit are far different than within the skull [29]. The relationship of the intracranial to orbital CSFp is still poorly understood, and therefore, we are limited to accepting at least the intracranial contribution of this pressure.

While this retrospective study confirms the correlation between CSFp and OAG, future prospective studies measuring IOP and CSFp in tandem would be useful to provide increased evidence for the TLPD theory in OAG.

## 6. Conclusions

In this case-control study, OAG patients had significantly lower CSFp than controls (9.9 ± 3.0 mm·Hg compared with 12.1 ± 3.6 mm·Hg, *p* < 0.0001), consistent with an association between OAG and reduced CSFp. The association of increased CSFp in OHT was not confirmed in this investigation. The TLPD theory supplements existing mechanical and vascular theories to describe a common mechanism for glaucomatous damage.

## Figures and Tables

**Table 1:** Reasons for exclusion.

	OAG	OAG control	OHT	OHT control
Identified	382	1481	215	2450
Excluded	296	1367	171	2363

Reason				
No recorded CSFp	153	709	74	1059
Not randomly selected for review	0	237	0	564
Did not meet eye criteria	83	93	38	112
Abnormal CSF, PMH, PSH	60	328	59	628

Total included	86	114	44	87

CSFp: cerebrospinal fluid pressure; CSF: cerebrospinal fluid; PMH: past medical history; PSH: past surgical history. The table summarizes reasons for exclusion of patients from each group.

**Table 2:** Validation of 2007–2019 data.

	OAG control	OAG	*p* value	NTG only	*p* value	OHT control	OHT	*p* value
	(*n* = 48)	(*n* = 29)		(*N* = 7)		(*N* = 48)	(*N* = 17)	
Biological sex (M)	27 (56.3%)	17 (58.6%)	1.0000	5 (71.4)	0.69	24 (50%)	8 (47.1%)	1.0000
Ethnicity, *n* (%)			0.40		1.0000			1.0000
Hispanic or Latino	0 (0.0%)	0 (0.0%)		0 (0.0%)		1 (2.1%)	0 (0.0%)	
Not Hispanic or Latino	43 (89.6%)	28 (96.6%)		7 (100.0%)		42 (87.5%)	15 (88.2%)	
Unknown	5 (10.4%)	1 (3.4%)		0 (0.0%)		5 (10.4%)	2 (11.8%)	

Race, *n* (%)			0.29		1.0000			0.35
Asian	0 (0.0%)	1 (3.4%)		0 (0.0%)		0 (0.0%)	1 (5.9%)	
White	46 (95.8%)	28 (96.6%)		7 (100.0%)		43 (89.6%)	15 (88.2%)	
Unknown/not reported	2 (4.2%)	0 (0.0%)		0 (0.0%)		3 (6.3%)	1 (5.9%)	
Other	0 (0.0%)	0 (0.0%)		0 (0.0%)		2 (4.2%)	0 (0.0%)	

Age in years at LP, mean (SD)	68.2 (8.1)	731 (12.4)	0.04^*∗*^	76.3 (11.4)	0.05^*∗*^	59.0 (13.7)	59.4 (15.4)	0.85
BMI mean (SD)	28.8 (5.5)	26.8 (5.9)	0.17	26.3 (4.4)	0.23	29.6 (7.3)	27.8 (7.2)	0.39
LP indication			<0.01^*∗*^		0.09			0.71
Headache	2 (4.2%)	2 (6.9%)		0 (0.0%)		6 (12.5%)	6 (35.3%)	
Peripheral neuropathy	10 (20.8%)	1 (3.4%)		0 (0.0%)		10 (20.8%)	4 (23.5%)	
Radiculopathy	5 (10.4%)	2 (6.9%)		0 (0.0%)		4 (8.3%)	1 (5.9%)	
IRB-approved research study	12 (25.0%)	13 (44.8%)		6 (85.7%)		10 (20.8%)	2 (11.8%)	
Altered mental status	0 (0.0%)	2 (6.9%)		0 (0.0%)		0 (0.0%)	0 (0.0%)	
Normal pressure hydrocephalus	0 (0.0%)	2 (6.9%)		1 (14.3%)		0 (0.0%)	0 (0.0%)	
Generalized weakness	0 (0.0%)	2 (6.9%)		0 (0.0%)		3 (6.3%)	1 (5.9%)	
Stroke	0 (0.0%)	1 (3.4%)		0 (0.0%)		2 (4.2%)	0 (0.0%)	
Ataxia	5 (10.4%)	1 (3.4%)		0 (0.0%)		1 (2.1%)	1 (5.9%)	
Hearing loss	1 (2.1%)	0 (0.0%)		0 (0.0%)		1 (2.1%)	0 (0.0%)	
Pretransplant evaluation	3 (6.3%)	0 (0.0%)		0 (0.0%)		2 (4.2%)	1 (5.9%)	
Dementia	6 (12.5%)	1 (3.4%)		0 (0.0%)		8 (16.7%)	1 (5.9%)	
Hematological workup	4 (8.3%)	1 (3.4%)		0 (0.0%)		0 (0.0%)	0 (0.0%)	
Dizziness	0 (0.0%)	1 (3.4%)		0 (0.0%)		1 (2.1%)	0 (0.0%)	

CSF protein Mean (SD)	69.1 (47.0)	63.3 (23.5)	0.74			51.8 (25.6)	52.8 (20.1)	0.63
CSF glucose Mean (SD)	60.9 (17.0)	66.9 (15.7)	0.39			65.2 (27.7)	65.1 (18.9)	0.46
Systolic BP Mean (SD)	128.3 (16.2)	131.0 (18.2)	0.34	123.1 (19.5)	0.71	123.7 (15.1)	127.5 (18.3)	0.51
Diastolic BP Mean (SD)	74.2 (9.6)	72.7 (11.3)	0.52	71.1 (17.7)	0.60	73.8 (9.4)	76.8 (10.2)	0.28

Demographics are similar between cases and controls except for age in the OAG cases and OAG controls. Age was controlled for in all future multivariable analyses. Other patient characteristics from the lumbar puncture visit are similar between cases and controls. ^*∗*^Comparison achieved statistical significance (*p* < 0.05); ^*∗∗*^*n* = 47 for BMI in the OAG group.

## Data Availability

Access to data is restricted to maintain patient privacy per institutional policy. Data may be available upon request to the corresponding author (fautsch.michael@mayo.edu).

## References

[1] Weinreb R. N., Aung T., Medeiros F. A. (2014). The pathophysiology and treatment of glaucoma. *JAMA*.

[2] Shin D. H., Lee M. K., Briggs K. S., Kim C., Zeiter J. H., McCarty B. (1992). Intraocular pressure-related pattern of optic disc cupping in adult glaucoma patients. *Graefe Archive for Clinical and Experimental Ophthalmology*.

[3] Iwase A., Suzuki Y., Araie M. (2004). The prevalence of primary open-angle glaucoma in Japanese. *Ophthalmology*.

[4] Morgan W. H., Yu D. Y., Cooper R. L., Alder V. A., Cringle S. J., Constable I. J. (1995). The influence of cerebrospinal fluid pressure on the lamina cribrosa tissue pressure gradient. *Investigative Ophthalmology and Visual Science*.

[5] Berdahl J. P., Allingham R. R. (2010). Intracranial pressure and glaucoma. *Current Opinion in Ophthalmology*.

[6] Siaudvytyte L., Januleviciene I., Daveckaite A. (2015). Literature review and meta-analysis of translaminar pressure difference in open-angle glaucoma. *Eye*.

[7] Siaudvytyte L., Januleviciene I., Ragauskas A. (2014). The difference in translaminar pressure gradient and neuroretinal rim area in glaucoma and healthy subjects. *Journal of Ophthalmology*.

[8] Ren R., Jonas J. B., Tian G. (2010). Cerebrospinal fluid pressure in glaucoma: a prospective study. *Ophthalmology*.

[9] Berdahl J. P., Fautsch M. P., Stinnett S. S., Allingham R. R. (2008). Intracranial pressure in primary open angle glaucoma, normal tension glaucoma, and ocular hypertension: a case-control study. *Investigative Ophthalmology & Visual Science*.

[10] Berdahl J. P., Allingham R. R., Johnson D. H. (2008). Cerebrospinal fluid pressure is decreased in primary open-angle glaucoma. *Ophthalmology*.

[11] Wang N., Xie X., Yang D. (2012). Orbital cerebrospinal fluid space in glaucoma: the Beijing intracranial and intraocular pressure (iCOP) study. *Ophthalmology*.

[12] Liu H., Yang D., Ma T. (2018). Measurement and associations of the optic nerve subarachnoid space in normal tension and primary open-angle glaucoma. *American Journal of Ophthalmology*.

[13] Fazio M. A., Clark M. E., Bruno L., Girkin C. A. (2018). In vivo optic nerve head mechanical response to intraocular and cerebrospinal fluid pressure: imaging protocol and quantification method. *Scientific Reports*.

[14] Pircher A., Montali M., Berberat J., Remonda L., Killer H. E. (2017). Relationship between the optic nerve sheath diameter and lumbar cerebrospinal fluid pressure in patients with normal tension glaucoma. *Eye*.

[15] Li L., Li C., Zhong H., Tao Y., Yuan Y., Pan C.-W. (2016). Estimated cerebrospina fluid pressure and the 5-year incidence of primary open-angle glaucoma in a Chinese population. *PLoS One [Electronic Resource]*.

[16] Zhang Q., Zhang Y., Xin C. (2019). Determinants of maximum cup depth in non-glaucoma and primary open-angle glaucoma subjects: a population-based study. *Eye*.

[17] Jonas J. B., Wang N., Wang Y. X., You Q. S., Yang D., Xu L. (2014). Ocular hypertension: general characteristics and estimated cerebrospinal fluid pressure. the Beijing eye study 2011. *PLoS One*.

[18] Fleischman D., Berdahl J. P., Zaydlarova J., Stinnett S., Fautsch M. P., Allingham R. R. (2012). Cerebrospinal fluid pressure decreases with older age. *PLoS One*.

[19] Wostyn P., De Groot V., Van Dam D., Audenaert K., De Deyn P. P. (2013). Senescent changes in cerebrospinal fluid circulatory physiology and their role in the pathogenesis of normal-tension glaucoma. *American Journal of Ophthalmology*.

[20] Lehman R. A. W., Krupin T., Podos S. M. (1972). Experimental effect of intracranial hypertension upon intraocular pressure. *Journal of Neurosurgery*.

[21] Czorlich P., Kratzig T., Kluge N. (2019). Intraocular pressure during neurosurgical procedures in context of head position and loss of cerebrospinal fluid. *Journal of Neurosurgery*.

[22] Ferguson T. J., Knier C. G., Chowdhury U. R. (2020). Intraocular pressure measurement with pneumatonometry and a tonometer tip cover. *Ophthalmology and Therapy*.

[23] Kang S.-K., Murphy R. K. J., Hwang S.-W. (2016). Bioresorbable silicon electronic sensors for the brain. *Nature*.

[24] Bai W., Shin J., Fu R. (2019). Bioresorbable photonic devices for the spectroscopic characterization of physiological status and neural activity. *Nature Biomedical Engineering*.

[25] Berdahl J. P. (2013). Systemic parameters associated with cerebrospinal fluid pressure. *Journal of Glaucoma*.

[26] Lenfeldt N., Koskinen L. O. D., Bergenheim A. T., Malm J., Eklund A. (2007). CSF pressure assessed by lumbar puncture agrees with intracranial pressure. *Neurology*.

[27] Morgan W. H., Chauhan B. C., Yu D. Y., Cringle S. J., Alder V. A., House P. H. (2002). Optic disc movement with variations in intraocular and cerebrospinal fluid pressure. *Investigative Ophthalmology & Visual Science*.

[28] Morgan W. H., Yu D. Y., Alder V. A. (1998). The correlation between cerebrospinal fluid pressure and retrolaminar tissue pressure. *Investigative Ophthalmology & Visual Science*.

[29] Fleischman D., Kaskar O., Shams R. (2019). A novel porcine model for the study of cerebrospinal fluid dynamics: development and preliminary results. *Frontiers in Neurology*.

